# Plasmonic Metasurfaces for Switchable Photonic Spin–Orbit Interactions Based on Phase Change Materials

**DOI:** 10.1002/advs.201800835

**Published:** 2018-08-28

**Authors:** Ming Zhang, Mingbo Pu, Fei Zhang, Yinghui Guo, Qiong He, Xiaoliang Ma, Yijia Huang, Xiong Li, Honglin Yu, Xiangang Luo

**Affiliations:** ^1^ State Key Laboratory of Optical Technologies on Nano‐Fabrication and Micro‐Engineering Institute of Optics and Electronics Chinese Academy of Sciences Chengdu 610209 China; ^2^ Key Laboratory of Opto‐Electronic Technology and Systems of the Education Ministry of China Chongqing University Chongqing 400044 China; ^3^ University of Chinese Academy of Sciences Beijing 100049 China

**Keywords:** catenary optics, phase change materials, plasmonic metasurfaces, spin–orbit interactions, switchable photonic metadevices

## Abstract

Metasurfaces with intense spin–orbit interactions (SOIs) offer an appealing platform for manipulation of polarization and wavefront. Reconfigurable beam manipulation based on switchable SOIs is highly desired in many occasions, but it remains a great challenge since most metasurfaces lack the flexibility and the optical performance is fixed once fabricated. Here, switchable SOIs are demonstrated numerically and experimentally via the combination of plasmonic metasurfaces with phase change materials (PCMs). As a proof‐of‐concept, three metadevices possessing switchable SOIs are fabricated and investigated, which enable spin Hall effect, vortex beam generation, and holography when the PCM is in the amorphous state (corresponding to the “ON” state of SOI). When the PCM changes into the crystalline state (corresponding to the “OFF” state of SOI), these phenomena disappear. Experimental measurements show that a high polarization conversion contrast between “ON” and “OFF” states is obtained within a broadband wavelength range from 8.5 to 10.5 µm. The switchable photonic SOIs proposed here may provide a promising route to design reconfigurable devices for applications such as beam steering, dynamic holographic display, and encrypted optical communications.

## 1 Introduction

Recently, artificially structured metasurfaces have experienced a rapid development. Owing to the unique electromagnetic properties, metasurface allows for flexible manipulation of the polarization, phase, and amplitude of light, enabling many extraordinary applications such as sub‐diffraction imaging,^1^, ^2^, ^3^ anomalous beam deflection,^4^, ^5^, ^6^, ^7^ polarization and chirality manipulation,^8^, ^9^, ^10^ and exotic quantum effects.^11^, ^12^ These landmark achievements could open a door for the optical engineering at the subwavelength scale, i.e., for Engineering Optics 2.0.^13^ Benefiting from the spatially inhomogeneous and anisotropic building blocks and transverse gradient phase along the thin interface, metasurfaces are naturally taken as novel platforms for photonic spin–orbit interactions (SOIs).^14^, ^15^ SOIs describe the coupling between spin and orbital angular momentums of photons during the propagation of light,^16^ which enable numerous fundamental and applied studies, such as spin Hall effect of light (SHEL),^17^, ^18^ flat lensing,^19^, ^20^ optical holography,^21^, ^22^ vortex beam generation,^23^ polarization conversion, and virtual shaping.^24^, ^25^ Interestingly, asymmetric SOIs have been recently reported^26^, ^27^ and enable many multifunctional chiral devices.^28^ However, most existing SOI‐based devices possess a fixed functionality once fabricated, which hinder their widespread utilization in practice.

Phase change material (PCM) is a promising and earth‐abundant alternative to the next‐generation nonvolatile optical devices, offering a new avenue to realize the switchable photonic devices. GeSbTe (GST) alloys as typical phase change materials have been used for many years in optical disk storage^29^ and been introduced to reconfigurable photonic devices recently.^30^, ^31^ GST alloys can be switched repeatedly between amorphous and crystalline states or to an intermediate state by appropriate thermal,^32^ optical,^33^, ^34^ or electrical stimulus.^35^ Additionally, such materials exhibit high refractive index contrast between amorphous and crystalline states and low losses in the near‐ and middle‐infrared spectral range. With these extraordinary properties, GST alloys are ideal materials for switchable or reconfigurable devices, such as thermal emitters,^32^ Fresnel zone plates,^33^ and absorbers.^36^ Recently, several works^37^, ^38^, ^39^ have shown great potential of GST in dynamic wavefront manipulation and provide a new way to realize switchable SOIs.

In this work, we experimentally demonstrate the switchable SOIs in the mid‐infrared spectral range via the combination of plasmonic metasurface with the PCMs (GST). To verify the proposed approach for switchable SOIs, three metadevices based on a simple metal–insulator–metal (MIM) configuration are fabricated by microfabrication techniques. These designed metadevices turn on SOIs for SHEL, vortex beam generation, and optical holography when the GST is in amorphous state. When the GST layer turns into crystalline state, the SOIs are switched off and these phenomena disappear. These switchable metadevices are developed based on the SOIs accompanying Pancharatnam–Berry (PB) phase.^40^ Experimental spectral results show that the cross‐polarization reflectance of the unit cell approaches 60% in amorphous state and turns to approximately zero in crystalline state in a broadband wavelength range from 8.5 to 10.5 µm. We believe that the proposed approach for switchable SOIs has great potential for reconfigurable photonic applications.

## 2 Design and Methods

**Figure 1:**
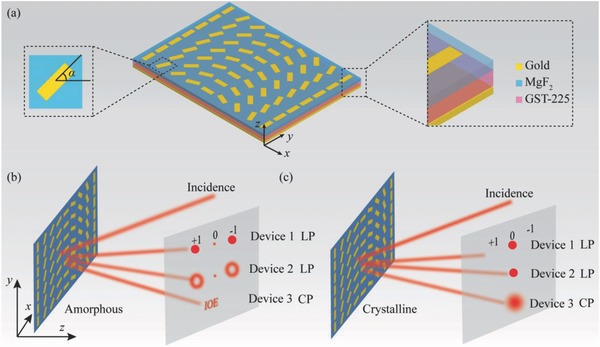
Schematic of the switchable photonic SOIs. a) Structure and materials. b,c) Optical performances of three designed metadevices when the GST layer is in b) amorphous and c) crystalline states.

A MIM configuration is adopted throughout this paper, as depicted in **Figure** **1**a. The insulator layer consisting of Ge_2_Sb_2_Te_5_ and MgF_2_ film is sandwiched by the bottom gold ground plane and the top array of subwavelength plasmonic gold antennas. The GST film acts essentially as a switchable dielectric medium that changes the optical response of the metadevices. MgF_2_ film is deposited on the top of the GST layer to protect it against oxidation in the atmosphere. Furthermore, the MgF_2_ layer serves as a refraction index matching layer between high‐index GST and low‐index air, resulting in a significant improvement of the polarization conversion efficiency.

Three functions are developed here to illustrate the switchable beam deflection, orbital angular momentum (OAM) generation, and holography. Each anisotropic metallic antenna can be taken as a local wave plate. The spatially variant orientation of these wave plates causes intense SOIs. In this case, if the right/left circular incident light $E_{i}^{\mathrm{R/L}}$ normally impinges on the metasurface from +*z*‐direction, the resulting reflected light **E**_r_ can be described as^16^, ^40^

(1) $$E_{r}=E_{i}^{\mathrm{R/L}}\cos\frac{\delta }{2}+i\sin\frac{\delta }{2}e^{\mp 2i\alpha }E_{i}^{\mathrm{L/R}}$$

where δ is the phase shift between the main axes of wave plate and the fast axis is oriented at an angle of α with respect to the *x*‐axis.

Equation (1) shows that, under normal incidence, a circularly polarized (CP) beam is scattered into waves of the similar polarization as that of the incident beam without phase change, and waves of the opposite circular polarization with a spin‐dependent phase change of Φ = 2*σα*, where σ = ± 1 denotes the state of circular polarization. Obviously, by controlling the local orientation of the fast axes of the metallic elements between 0 and π, one can realize phase variation covering the full 0‐to‐2π range while maintaining equal reflection amplitude. Note that the amplitude of opposite circular polarization depends on the phase retardation δ, a wave plate with a phase retardation of π is desired to realize 100% conversion efficiency.

Based on the SOIs and geometric phase, we designed three metadevices to demonstrate switchable functions. As shown in the Figure 1b, when the GST layer is in amorphous state, SOIs occur in the three metadevices. Under the illumination of linear polarization (LP), the devices 1 and 2 show an angular SHEL and generate two deflected vortex beams, respectively. Under the illumination of CP light, the third device generates one holographic image of abbreviation of Institute of Optics and Electronics (IOE). However, when the GST layer is switched into crystalline state, the SOIs‐enabled phenomena disappear and there is only a bright spot. In this case, all of the three devices behave as conventional reflective mirrors.

## 3 Results and Discussions

**Figure 2:**
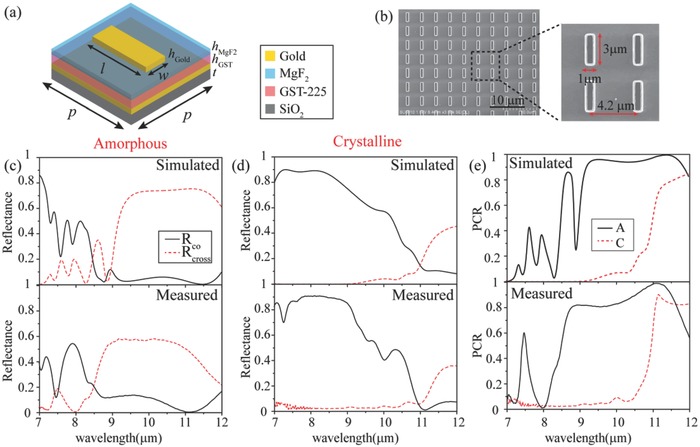
a) Schematic of the unit cell. b) SEM images of fabricated patch antennas array with the width *l* = 3 µm, length *w* = 1 µm, and *p* = 4.2 µm. c,d) The co‐polarization and cross‐polarization reflectance in c) amorphous and d) crystalline states. e) The PCR with respect to the wavelength in amorphous (A) and crystalline (C) states.

Although our switchable metadevices are designed and optimized for middle infrared (MIR) wavelength, specifically at λ = 9.6 µm, the proposed concept can be readily extended for operating at other desired wavelengths like visible and near infrared spectral range. The schematic of the unit cell of switchable photonic SOIs‐based devices is depicted in **Figure** **2**a. The thickness of GST *h*_GST_ is 600 nm to turn off the polarization conversion after crystallization. The thickness of MgF_2_ (*h*_MgF2_ = 150 nm) is chosen to be thick enough to block the contact between air and GST. The antennas array is composed rectangle patches with a length of *l* and a width of *w*, and the thickness of antennas (*h* = 100 nm) is chosen to be as thin as possible to reduce the fabrication difficulty. Figure 2b presents the scanning electron microscope (SEM) images of the fabricated antennas array. The length, width, and period of patch antenna are 3, 1, and 4.2 µm, respectively. More fabrication details are depicted in the Experimental Section and Section S2 in the Supporting Information.

Numerical simulations are performed using the finite element method (FEM) in a commercial software package CST Microwave Studio (more detailed setup of simulation is presented in the numerical simulation and Section S1, Supporting Information). The simulated results are shown in the upper part of Figure 2c,d. As illustrated in the figure, the reflectance of cross‐polarization is over 70% in a broad spectral range from 9 to 12 µm in the amorphous state, while the co‐polarized reflectance is approximately close to 0 (Here the circular polarization conversion of a common mirror, i.e., a left‐handed circular polarization (LCP) will be reflected as right‐handed circular polarization (RCP), is neglected for simplicity of discussion). When GST changes into crystalline state, the cross‐polarized reflectance turns to be less than 5% in 7–11 µm. The performance of the sample was characterized with a Fourier‐transform infrared spectrometer (Bruker Vertex 80) in reflection mode with an incident angle of 15°. In order to characterize the broadband performance, we utilized two linear polarizers at 45° with respect to the patch antenna instead (an explanation for the experimental setup is given in the Experimental Measurement in the Experimental Section). The measured results are shown in the bottom part of Figure 2c,d. In amorphous state, the cross‐polarized reflectance approaches to 60% and the co‐polarized reflectance is less than 10% in the spectral range from 8.5 to 11.5 µm. The sample was heated on a hotplate for 20 min to ensure the complete crystallization. In crystalline state, the cross‐polarized reflectance decreases to be less than 2%. Besides, we calculated the polarization conversion ratio (PCR), defined as PCR = *R*_cross_/(*R*_cross_ + *R*_co_), to characterize the working bandwidth. Figure 2e shows the simulated and measured PCR with respect to the wavelength in two states, respectively. The simulated results depict that the PCR is beyond 90% in amorphous state and below 10% in crystalline state in a broadband spectral range from 9 to 11.5 µm, indicating a broadband feature. The measured results also show the broadband feature whose PCR is beyond 80% in amorphous state and below 10% in crystalline state in a broadband spectral range from 8.5 to 10.5 µm.

As illustrated in Figure 2, we should note that the cross‐polarized reflectance in experiment is lower than the simulation accompanying a blueshift of working bandwidth, which can be attributed to the following reasons. 1) The imperfection of the fabrication, including the thickness of stack layers and the geometric dimensions of patch antennas, modifies the performance of the sample. The deviations of thickness and geometric dimensions will cause the shift of working band. 2) The complex permittivity of materials used in the simulation may have certain deviations from experiment. The actual extinction coefficients of GST and MgF_2_ may be larger than the simulated parameters, resulting in higher absorption and lower cross‐ and co‐polarized reflectance. Overall, the measured results agree well with the simulated results.

**Figure 3:**
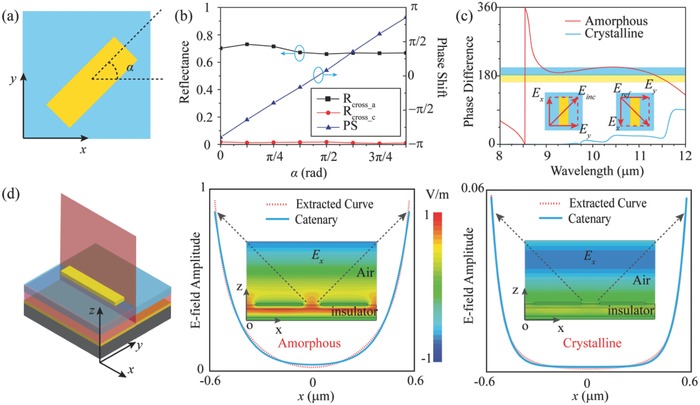
a) Top view of the unit cell with an orientation angle of α. b) Simulated cross‐polarization reflectance and phase shift as a function of α in two states. c) The calculated phase difference between the two orthogonal polarizations in two states. d) 3D view of the unit cell and the electric field distributions in the red cut plane. The right two panels show the extracted electric field amplitude (red dotted line) and fitted catenary curve (blue solid line) between two adjacent patches for amorphous state (left) and crystalline state (right) at 9.6 µm, respectively. The insets are the distributions of electric field **E** *_x_* in the unit cell under the illumination of LCP.

To present the physical mechanism of switchable SOIs, we construct and analyze the unit cell with different orientation angle α (as depicted in **Figure** **3**a). Figure 3b shows the relationship of reflectance and phase shift with respect to the orientation angles. *R*_cross_a_ and PS represent the cross‐polarized reflectance and phase shift at 9.6 µm in amorphous state, and in this case cross‐polarized reflectance remains approximately the same as the angle α varies. The calculated phase shift shows a good agreement with PB phase. We can also see that the unit cells keep low cross‐polarized reflectance in crystalline state from red line of R_cross_c_.

Furthermore, to explore the physical mechanism of different responses of the unit cell in two states, the reflective phase difference between two LP components along the *x* and *y* directions are calculated as depicted in Figure 3c. For the unit cell illuminated by a normally incident LP light with a polarization angle of 45°, a phase difference of π with a slight variation in the range of 0.9π to 1.1π (the bandwidth is defined by 10% change in ΔΦ) is achieved from 8.8 to 11.5 µm in amorphous state, which is a requirement to convert a CP light to its cross‐polarization state. This result agrees well with the aforementioned measured results and the conclusions in previous works.^9^, ^41^ However, the phase difference between two orthogonal components in crystalline state cannot meet the condition of polarization conversion, which results in a normal specular reflection.

In an effort to get a more clear visualization of this effect, we illustrate the instantaneous electric field distributions under normal incidence. Figure 3d shows the distributions of electric field **E** *_x_* at the resonant wavelength of 9.6 µm in the *yz* plane. The electric field in amorphous state (left) is obviously enhanced at the metal‐insulator interface and coupled with that in the adjacent elements, and interestingly the electric field profile between the adjacent elements can be described by the well‐known catenary curves (the fitted curves are shown in blue solid lines in Figure 3d and the generalized catenary model is given in Section S3, Supporting Information). Such field distribution stems from the near field interaction between adjacent unit cells.^42^, ^43^ By tuning the coupling strength of catenary optical fields, ultrabroadband SOI can be enabled or suppressed. In crystalline state, the refractive index of GST gets very large, and the transversal coupling is suppressed, resulting in a weak anisotropy and thus low polarization conversion efficiency.

## 4 Characterization of Metadevices

**Figure 4:**
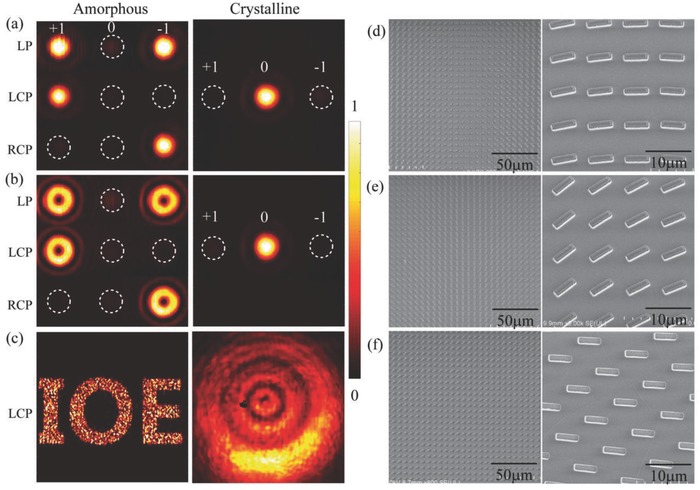
The measured intensity distributions and SEM images of three metadevices with switchable SOIs. The intensity patterns for a) SHEL, b) vortex generation, and c) holography in amorphous and crystalline states. The constant background produced by thermal radiation has been removed. d–f) SEM images of the three metadevices.

In order to demonstrate the versatility of our proposed approach, we fabricated and characterized three typical flat metadevices. The schematic diagram of the photonic SOIs of three metadevices is presented in the Figure 1. Experimental results in different states (amorphous and crystalline states) and SEM images of the three metadevices are shown in **Figure** **4**.

The first device is fabricated to demonstrate SHEL. The phase gradient induced by a linear change of orientation with the coordinates produces a helicity‐dependent transverse wave vector. Thus, the anisotropic metasurface deflects right‐ and left‐hand polarized beams to opposite directions, which can be considered as an anisotropy‐induced SHEL. The designed metadevice consists of periodic arrays of 16 unit cells with an incremental rotation angle of π/16. The left panel of Figure 4a shows the measured reflected diffraction patterns at the wavelength of 9.6 µm when the device is in amorphous state. The central dim spot (0th order) is attributed to the unmodulated spin component of reflected beam, while +1st‐ and −1st‐order bright spots stem from the SHEL of two converted spin components. Under the illumination of LP light, the measured efficiencies of the +1st‐, 0th‐, and −1st‐order diffractions are 26.1%, 2.4%, and 26.1%, respectively. Besides, under the illumination of LCP or RCP, it is observed that a majority of the reflected light concentrated in the +1st‐ or −1st‐order, thus the metadevice works as an anomalous deflector. When this device is heated for 20 min at 200 °C, the GST layer turns into crystalline state. In this case, the majority of reflected light is concentrated in the 0th‐order whatever the polarization of incident light is, so the SHEL is “switched off” and the metadevice behaves as a mirror‐like reflector.

The second device is created as a vortex beam generator, which is crucial for many classic and quantum applications. To facilitate the measurement, an azimuthal phase dependence exp(*ilφ*) is superimposed on the above mentioned deflector, where φ is the azimuthal angle and the *l* is the topological charge. As illustrated in the Figure 4b, the donut‐shape patterns present at +1st‐ and −1st‐order (LP incidence) and at +1st/−1st‐order (LCP/RCP incidence) that features the generated vortices. When the device turns into crystalline state, the donut‐shape patterns vanished and there is only a bright spot at the 0th‐order. The device exhibits the capability of allowing conversion between the orbital angular momentum and spin angular momentum. Furthermore, a sample with the topological charge *l* = −6 is fabricated and the topological charge is identified by interference with spherical beam in two states to demonstrate the potential for encrypted optical communications, as shown in the Figure S4 (Section S4, Supporting Information).

The last device is a hologram that generates one holographic image of abbreviation of the IOE. The holographic image is designed based on point‐source algorithm. The left panel of Figure 4c displays the measured reflected intensity pattern under the illumination of a light beam with pure circular polarization (RCP or LCP) in amorphous state. As the metadevice turns into crystalline state, the designed holographic pattern disappeared and there is only a bright spot. It could be considered that the information carried by the device is concealed or the device present different holographic pattern in two states. Such performance may provide a new route to dynamic hologram generation and encrypted information storage.

**Figure 5:**
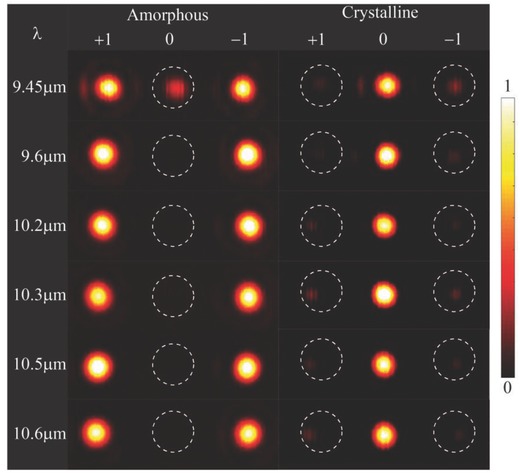
The measured reflected patterns produced by deflector in amorphous state (left) and in crystalline state (right) at different wavelengths. The constant background produced by thermal radiation has been removed.

To further characterize the broadband feature of the metadevices, we measured the reflected patterns of deflector under the illumination of LP light at several wavelengths ranging from 9.45 to 10.6 µm (available wavelength range of our laser) in two states. **Figure** **5** depicts the measured reflected diffraction patterns at several wavelengths in two states. It is obvious that the power of reflected light is concentrated in the ± 1st‐order for amorphous state while concentrated in the 0th‐order when the samples are crystallized in the whole range. The efficiencies of the +1st‐, 0th‐ and −1st diffraction orders were not measured here due to the instability of laser power at these wavelengths (9.45, 9.6, 10.2, 10.3, 10.5, and 10.6 µm).

The experimental results validated that the functions of metadevices can be switched between amorphous and crystalline states. The reamorphization of GST can be achieved by several methods, such as thermal annealing, electrical stimulus and laser pulse illumination. However, the fact that the reamorphization temperature of the GST (≈640 °C) is beyond the melting point of 100 nm thick Au (≈300 °C) hinders the reconfiguration of our devices by thermal annealing process. Nevertheless, with proper design, electrical stimulus or laser pulses can be utilized to realize the reconfiguration of metadevices.^33^, ^34^, ^35^ Two possible methods (one is based on phase‐change electronic memory and another is based on laser‐direct writing) are illustrated in Section S5 of Supporting Information. The typical switching time is several nanoseconds for electrical stimulus^44^ and even merely tens of femtoseconds for laser pulses.^34^

## 5 Conclusion

In summary, we have successfully combined the phase change materials with plasmonic metasurfaces to realize the switchable spin–orbit interactions working in the mid‐infrared spectral range. The SOIs‐enabled phenomena are achieved by using the MIM structure and catenary optical fields. Based on the ingenious design, we can realize switching of “ON” and “OFF” states. Spectral characterization results show a high polarization conversion contrast between the two states accompanying a broadband working bandwidth, which agrees well with simulated results. Three metadevices are designed and fabricated to verify the approach for switchable SOIs. The simple fabrication process provides great potential for practical applications in beam steering, encrypted optical communications, and dynamic holographic display.

## 6 Experimental Section

*Numerical Simulation*: The FEM in a commercial software package CST microwave studio was used to calculate the co‐ and cross‐polarized reflectance of the unit cell. The permittivity of gold was described by Drude model, and the dielectric constants of MgF_2_ and GST were acquired experimentally by spectroscopic ellipsometer (SENTECH SE850 and SENDIRA) (more details are shown in Section S1, Supporting Information). All the models described in this paper were meshed using triangular elements with a maximum size of λ_0_/(50*n*) in dielectric materials and of λ_0_/100 in gold. The unit cell boundary was employed in the *x* and *y* directions. For the *z*‐direction, the perfectly matched layer boundary was applied. The incident light was a 45° polarized LP wave with an incident angle of 15° due to the minimum measured angle of Fourier transform infrared spectroscopy (FTIR) in the reflection mode.

*Device Fabrication*: The metadevices (an overall area of 1 × 1 cm^2^) were fabricated as follows. First, the (bottom) gold, GST, MgF_2_, and (top) gold layers were deposited onto a clean silicon dioxide substrate in sequence. The gold and GST layers were deposited using magnetron sputtering in an Ar atmosphere (base pressure: 1.0 × 10^−3^ Pa, Ar pressure: 3 × 10^−2^ Pa). The MgF_2_ layer was deposited by electron beam evaporation. Next, plasmonic antennas were patterned on top of the layered structure by using laser direct writing lithography to create an appropriate photoresist mask, which was a 1 µm thick layer of positive photoresist (AZ1500). More specifically, the photoresist was spin coated (2500 rpm) onto the sample and baked at low temperatures (100 °C) for 5 min to harden the resist but avoid crystallization of the GST layer (the as‐deposited GST state is amorphous and the temperature of crystallization is 160 °C). Then, the photoresist pattern was transferred to the top gold layer by the ion beam etching. The resist mask was removed by reactive ion etching for 30 min. Finally, the metadevices were obtained with switchable SOIs. Morphologic analysis of the samples was carried out using optical microscopy and SEM. An explanatory figure showing the fabrication procedures can be found in Section S2 in the Supporting Information.

**Figure 6:**
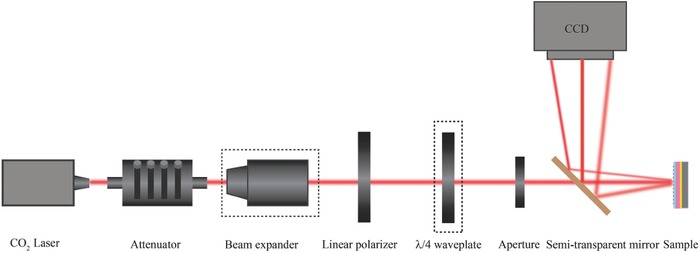
Schematic illustration of the measurement setup.

*Experimental Measurement*: **Figure** **6** displays the schematic illustration of the measurement setup. A CO_2_ laser was utilized as the light source. After passing through an adjustable attenuator, the optical beam was sent through a linear polarizer, followed by an adjustable aperture, and then illuminated on the sample from top of the metadevices. A beam splitter was utilized in front of the sample to guide reflected diffraction patterns to the infrared charge‐coupled devices (CCD) (384 × 288 pixels, UA330, Guide‐Infrared Inc.), whose size of each pixel is 25 µm × 25 µm. A quarter waveplate and a beam expander were inserted into the experimental setup to measure the holographic images and identify topological charge of vortex beam (Section S4, Supporting Information).

To measure the co‐polarized and cross‐polarized reflectance, the Fourier transform infrared spectroscopy was used with reflection accessory. Since a relatively narrow bandwidth of commercial quarter‐wave plate possessed challenges to generate broadband CP light in experimental setup, two linear polarizers were utilized instead.

## Conflict of Interest

The authors declare no conflict of interest.

## Supporting information

Supplementary
