# Supplementary material for: Plasmonic Metasurfaces for Switchable Photonic Spin–Orbit Interactions Based on Phase Change Materials
Source: Adv Sci (Weinh). 2018 Aug 28;5(10):1800835. doi: 10.1002/advs.201800835 (PMC6193175; doi:10.1002/advs.201800835)
Supplement: Supplementary file 1 — Supplementary [file ADVS-5-1800835-s001.pdf]

Copyright WILEY-VCH Verlag GmbH & Co. KGaA, 69469 Weinheim, Germany, 2018.

ADVANCED SCIENCE
Open Access

Supporting Information

for *Adv. Sci.,* DOI: 10.1002/advs.201800835

Plasmonic Metasurfaces for Switchable Photonic Spin–Orbit Interactions Based on Phase Change Materials

*Ming Zhang, Mingbo Pu, Fei Zhang, Yinghui Guo, Qiong He, Xiaoliang Ma, Yijia Huang, Xiong Li, Honglin Yu, and Xiangang Luo**

Supporting Information

Plasmonic Metasurfaces for Switchable Photonic Spin-orbit Interactions Based on Phase Change Materials

*Ming Zhang, Mingbo Pu, Fei Zhang, Yinghui Guo, Qiong He, Xiaoliang Ma, Yijia Huang, Xiong Li, Honglin Yu, Xiangang Luo**

Dr. Ming Zhang, Prof. Mingbo Pu, Dr. Fei Zhang, Dr. Yinghui Guo, Dr. Qiong He, Prof. Xiaoliang Ma, Prof. Xiong Li, Dr. Yijia Huang, Prof. Xiangang Luo
State Key Laboratory of Optical Technologies on Nano-Fabrication and Micro-Engineering, Institute of Optics and Electronics, Chinese Academy of Sciences, Chengdu 610209, China
E-mail: lxg@ioe.ac.cn
Dr. Ming Zhang, Dr. Fei Zhang, Prof. Honglin Yu
Key Laboratory of Opto-electronic Technology and Systems of the Education Ministry of China, Chongqing University, Chongqing 400044, China
Dr. Yijia Huang
University of Chinese Academy of Sciences, Beijing 100049, China

S1. Simulations: The permittivity of the materials

The permittivity of MgF_2 and $Ge_2Sb_2Te_5$ films used in the simulations are plotted in **Figure S1** and **Figure S2**, respectively. These values were measured by spectroscopic ellipsometer (SENTECH SE850 and SENDIRA) for the visible and infrared spectral range. We deposited 50nm-thick GST layer on a silicon substrate to measure its permittivity (amorphous state). Then, the sample was crystallized on a bake plate for 30 min, where the temperature was set as 200 ℃. From Figure S1, we can see that the real part of the permittivity shows a large difference between the amorphous and crystalline states within a broad mid-infrared wavelength region ($\varepsilon_1 \approx 12$ in amorphous state and $\varepsilon_1 \approx 25$ in crystalline state at least from 4 to 16 μm). The GST possesses the low-loss dielectric properties because the imaginary parts of permittivity at both states are small compared to the real part ($\mathrm{Im}(\varepsilon)/\mathrm{Re}(\varepsilon) < 0.1$). Based on this unique dielectric properties, GST can be used to achieve high-efficiency metadevices. The permittivity of MgF_2 film is measured by depositing on silicon substrate with a thickness of 50 nm. As shown in the Figure S2, the real part of permittivity is close to 1 and the imaginary part is close to 0, which can work as the refraction index

matching layer. Due to this unique character, adding MgF_2 layer on the top of GST layer can improve the performance of metadevices. Furthermore, we also measure the permittivity of GST at visible and near-infrared (NIR) spectral range, and the properties of permittivity are similar to MIR region. So we can extend this method to other region by adjusting the geometric parameters of the unit cell. The complex permittivity of gold is described by Drude model[1]:

$$\varepsilon_{gold} = \varepsilon_{\infty} - \frac{\omega_p^2}{\omega(\omega + i\gamma)}$$

The plasma frequency ω_p and collision frequency γ are chosen to be 1.32×10^{16} rad/s and 131.8 THz, respectively. ε_{∞} is the relative permittivity when frequency is infinite and is set as 9.1.

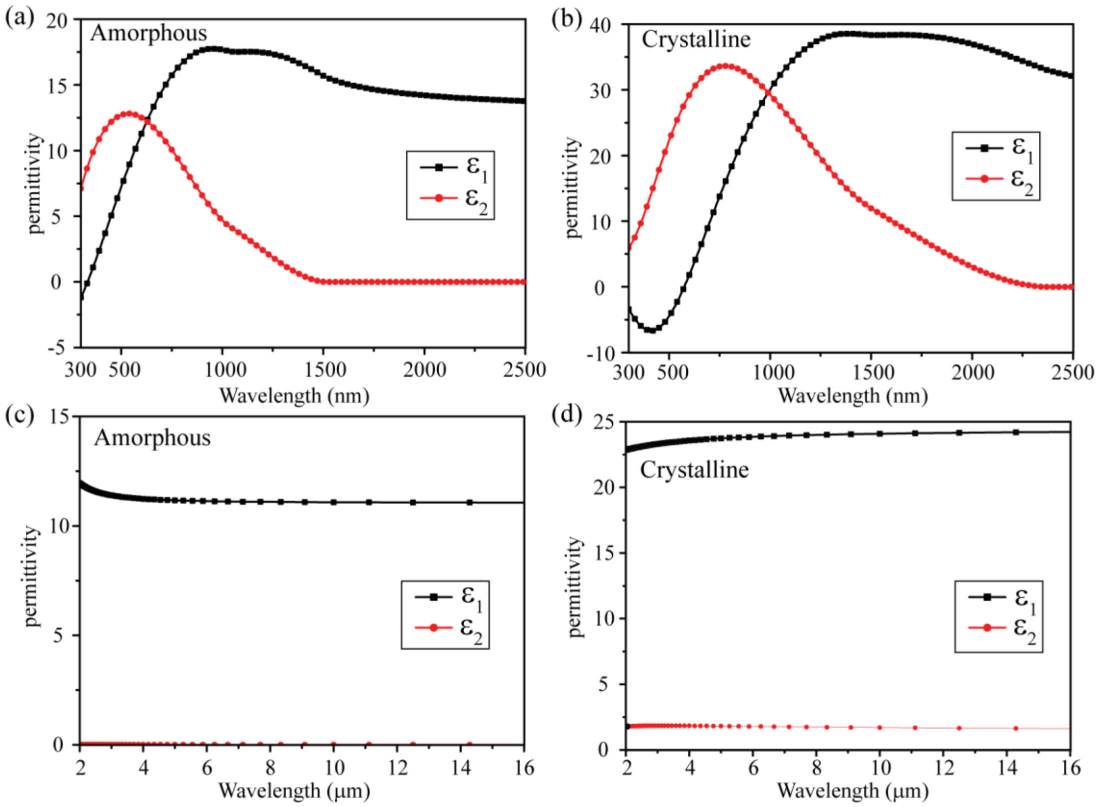

Figure S1. The real and imaginary parts of the measured permittivity of the GST film for both amorphous and crystalline states. a),b) Wavelength range from 0.3 to 2.5μm measured by SENTECH SE850. c),d) Wavelength range from 2.5 to 16μm measured by SENTECH SENDIRA.

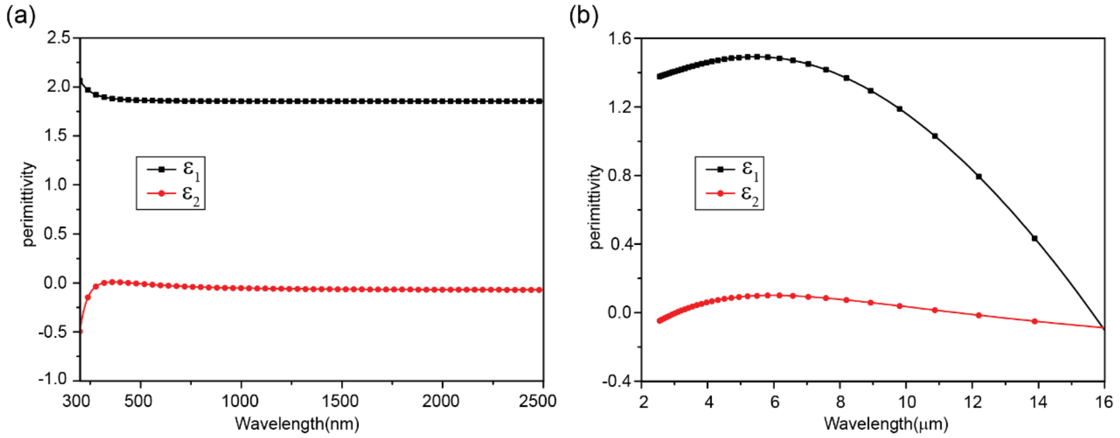

Figure S2. Measured permittivity of MgF_2 film. a),b) The real and imaginary part in the visible and near-infrared spectral range. c),d) Mid-infrared spectral range.

S2. Fabrication

Figure S3 presents a detailed sketch map of the fabrication process described in the main text.

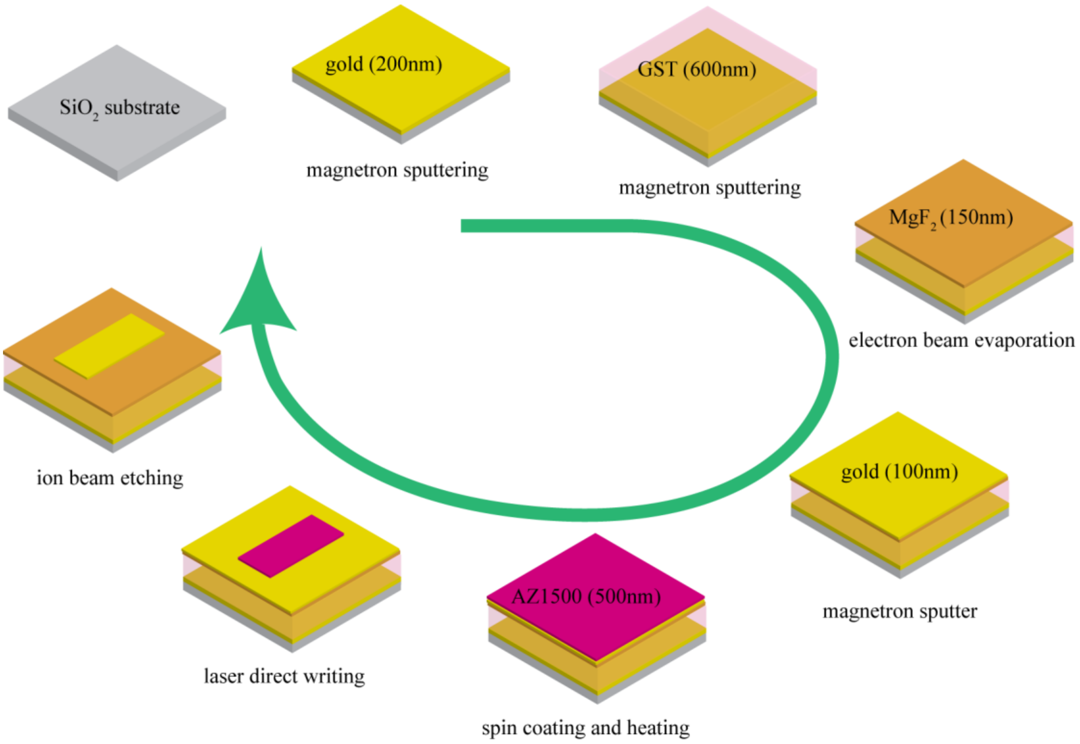

Figure S3. Fabrication process of metadevices.

S3. Analytical method

Catenary model. The generalized catenary model can be expressed as:

$$C(x) = a\cosh\left(\frac{x}{b} + \right) \quad (1)$$

where *a*,*b* and *c* are the variables in the function. The fitting process is achieved by employing the build-in curve fitting tool in Matlab. The retrieved parameters as well as the coefficient of determination (R-square) of each fitting for Figure 3d are shown in **Table S1**.

Table S1. Retrieved parameters and R-square of each fitting.

	a	b	c	R-square
Amorphous	0.002659	0.065	4.84	0.9977
Crystalline	0.0005871	-0.06484	1.301	0.9980

S4. Characterization of the vortex generator

To measure the topological charge of the vortex generator, we fabricated a device with two parts of phase. While one part is used to generate the OAM and the other is to generate a spherical wavefront. The interference between the helical wavefront and the spherical wavefront occurs in output cross-polarization light when illuminated with CP light. The vortex beam can be expressed as[2, 3]:

$$\phi_1(x,y) = l\varphi + \phi_0, \tag{2}$$

where l is topological charge and φ is the azimuthal angle, ϕ_0 is the spherical phase distribution corresponding to a focal length of f:

$$\phi_0(x,y) = \frac{2\pi}{\lambda}(\sqrt{f^2 + x^2 + y^2} - f). \tag{3}$$

Figure S4 shows the reflected patterns of vortex generator in two states and its optical microscopy image. The topological charge of l = -6 can be identified through the interference between the vortex beam and focusing spherical wavefront in amorphous state. When the vortex generator is in crystalline state, the reflected patterns turn to be mixed and in disorder, that is, the information carried by the device is missing. The metadevice shows great potential for encrypted optical-communication.

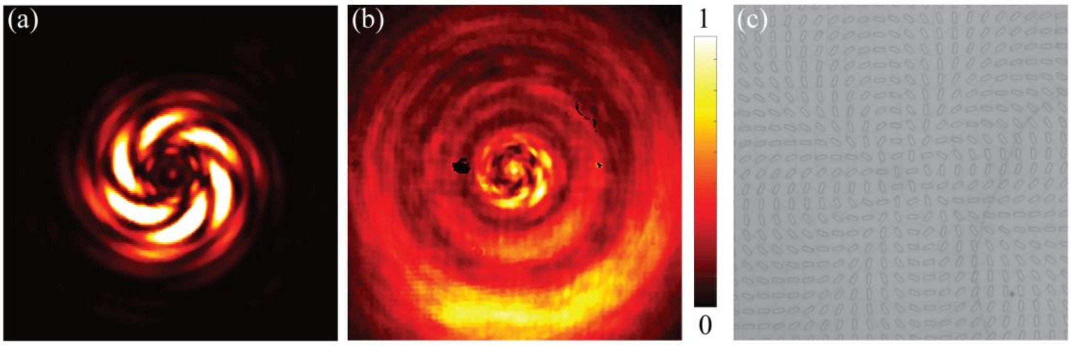

Figure S4. The reflected patterns of the vortex generator in a) amorphous and b) crystalline states. c) The optical microscopy image.

S5. Reconfiguration of the metadevices

To realize the reconfiguration of metadevices, several relatively mature techniques can be adopted. Two possible methods are illustrated in the Figure S5. Figure S5(a) gives a method based on phase-change electronic memory. The metasurfaces are made on the TiN electrodes (for converting the electronic energy to thermal energy). By applying a specific electrical stimulus, the phase state can be changed, thus realizing the reconfiguration of metadevice. The second method is based on laser-direct writing, which is illustrated in the Figure S5 (b). The reamorphization of GST layer can be achieved by a single laser pulse with high power intensity, and the crystallization of GST layer can be achieved by pulse sequence with lower power intensity[4-6]. Additionally, different from thermal annealing, the laser pulses do not damage the gold patch-antenna. Utilizing a scan femtosecond (fs) laser, the pulse duration can be controlled by adjusting the scan speed.

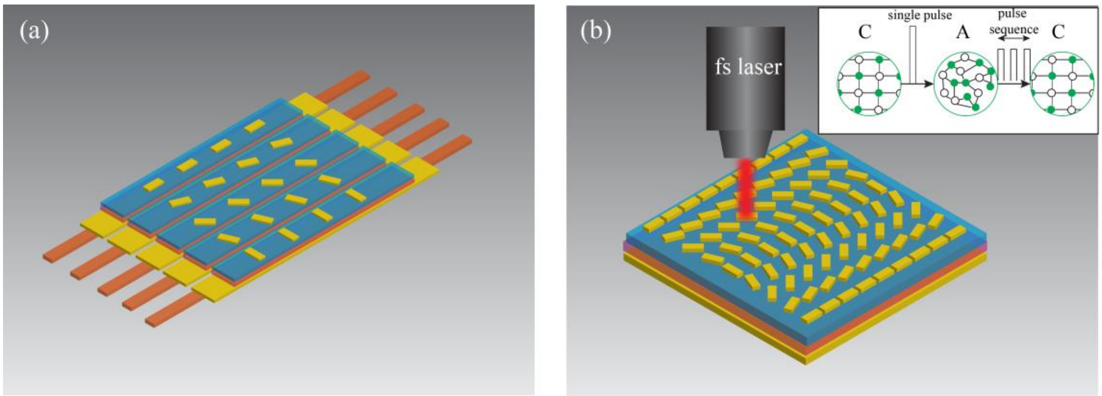

Figure S5. Schematics for realization of the reconfiguration of metadevices. a) Phase-change electronic memory. b) Laser-direct writing.

Reference

[1] N. Liu, M. Mesch, T. Weiss, M. Hentschel, H. Giessen, *Nano Lett.* **2010**, *10*, 2342.
[2] X. Chen, L. Huang, H. Mühlenbernd, G. Li, B. Bai, Q. Tan, G. Jin, C. W. Qiu, Z. Shuang, T. Zentgraf, *Nat. Commun.* **2012**, *3*, 1198.
[3] M. Pu, X. Li, X. Ma, Y. Wang, Z. Zhao, C. Wang, C. Hu, P. Gao, C. Huang, H. Ren, *Sci. Adv.* **2015**, *1*, e1500396.
[4] A. K. U. Michel, P. Zalden, D. N. Chigrin, M. Wuttig, A. M. Lindenberg, T. Taubner, *ACS Photonics* **2014**, *1*, 833.
[5] Q. Wang, E. T. F. Rogers, B. Gholipour, C. M. Wang, G. Yuan, J. Teng, N. I. Zheludev, *Nat. Photonics* **2015**, *10*, 60.
[6] C. R. D. Galarreta, A. M. Alexeev, Y. Y. Au, M. Lopez-Garcia, M. Klemm, M. Cryan, J. Bertolotti, C. D. Wright, *Adv. Funct. Mater*. **2018**, *28*, 1704993.
